# Supplementary material for: Entangled photons from on-chip slow light
Source: Sci Rep. 2014 Jan 28;4:3913. doi: 10.1038/srep03913 (PMC3904141; doi:10.1038/srep03913)
Supplement: Supplementary Information [file srep03913-s1.pdf]

Supplementary Information

Entangled photons from on-chip slow light

Hiroki Takesue[1,*], Nobuyuki Matsuda[1,2], Eiichi Kuramochi[1,2], and Masaya Notomi[1,2]

[1]NTT Basic Research Laboratories, NTT Corporation, 3-1 Morinosato Wakamiya, Atsugi, Kanagawa, 243-0198, Japan

[2]Nanophotonics Center, NTT Corporation, 3-1 Morinosato Wakamiya, Atsugi, Kanagawa, 243-0198, Japan

[*]Corresponding author (takesue.hiroki@lab.ntt.co.jp)

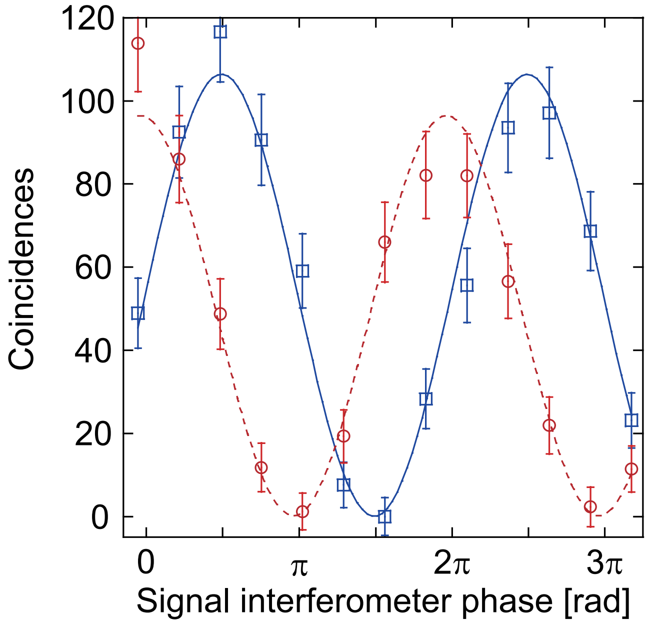

Supplementary Figure S1: Two-photon-interference fringes with accidental coincidences subtracted from the data shown in Fig. 3 (c). Squares: idler interferometer phase at 0, circles: $\pi/2$. Coincidences were counted for 10 million start pulses. The visibilities of the fitted curves were $99.9 \pm 7.6\%$ (idler interferometer phase 0) and $99.6 \pm 7.4\%$ ($\pi/2$).

Although the number of accidental coincidences does not in theory exceed that of raw coincidences, we observed a negative count when we subtracted the accidental coincidences (at the signal interferometer phase 1.56π and the idler interferometer phase 0), which was caused by the statistical fluctuation in the measured values of accidental coincidences at each phase. Since such a negative coincidence count is unphysical, we set the corresponding value at 0 and obtained a fitted curve.
